# Establishment of prognostic risk model and drug sensitivity based on prognostic related genes of esophageal cancer

**DOI:** 10.1038/s41598-022-11760-1

**Published:** 2022-05-14

**Authors:** Jingjing Dai, Abdusemer Reyimu, Ao Sun, Zaxi Duoji, Wubi Zhou, Song Liang, Suxia Hu, Weijie Dai, Xiaoguang Xu

**Affiliations:** 1 Department of Hematology, The Affiliated Huaian No.1 People’s Hospital of Nanjing Medical University, Huai’an, 223300 Jiangsu People’s Republic of China; 2 Medical College, Anhui University of Science and Technology, Huainan, 232001 Anhui People’s Republic of China; 3 Class 11, grade 2018, Clinical Medicine, Nanjing Medical University, Nanjing, 223300 Jiangsu People’s Republic of China; 4 Research Center of High Altitude Medicine, Naqu, Tibet, China, People’s Hospital of Naqu Affiliated to Dalian Medical University, Tibet, 852000 People’s Republic of China; 5 Department of Pathology, The Affiliated Huaian No.1 People’s Hospital of Nanjing Medical University, Huai’an, 223300 Jiangsu People’s Republic of China; 6 Department of Medical Laboratory, Second branch, The Affiliated Huaian No, People’s Hospital of Nanjing Medical University, Huai’an, 223300 Jiangsu People’s Republic of China; 7 Department of Medical Laboratory, Huainan First People’s Hospital, The First Affiliated Hospital of Anhui University of Science and Technology, Huainan, Anhui People’s Republic of China; 8 Department of Endoscopy Center, The Affiliated Huaian No.1 People’s Hospital of Nanjing Medical University, Huai’an, 223300 Jiangsu People’s Republic of China

**Keywords:** Cancer, Genetics, Immunology, Molecular biology, Biomarkers, Diseases, Gastroenterology, Medical research, Molecular medicine, Oncology, Risk factors

## Abstract

At present, the treatment of esophageal cancer (EC) is mainly surgical and drug treatment. However, due to drug resistance, these therapies can not effectively improve the prognosis of patients with the EC. Therefore, a multigene prognostic risk scoring system was constructed by bioinformatics analysis method to provide a theoretical basis for the prognosis and treatment decision of EC. The gene expression profiles and clinical data of esophageal cancer patients were gathered from the Cancer Genome Atlas TCGA database, and the differentially expressed genes (DEGs) were screened by R software. Genes with prognostic value were screened by Kaplan Meier analysis, followed by functional enrichment analysis. A cox regression model was used to construct the prognostic risk score model of DEGs. ROC curve and survival curve were utilized to evaluate the performance of the model. Univariate and multivariate Cox regression analysis was used to evaluate whether the model has an independent prognostic value. Network tool mirdip was used to find miRNAs that may regulate risk genes, and Cytoscape software was used to construct gene miRNA regulatory network. GSCA platform is used to analyze the relationship between gene expression and drug sensitivity. 41 DEGs related to prognosis were pre-liminarily screened by survival analysis. A prognostic risk scoring model composed of 8 DEGs (APOA2, COX6A2, CLCNKB, BHLHA15, HIST1H1E, FABP3, UBE2C and ERO1B) was built by Cox regression analysis. In this model, the prognosis of the high-risk score group was poor (*P* < 0.001). The ROC curve showed that (AUC = 0.862) the model had a good performance in predicting prognosis. In Cox regression analysis, the comprehensive risk score can be employed as an independent prognostic factor of the EC. HIST1H1E, UBE2C and ERO1B interacted with differentially expressed miRNAs. High expression of HIST1H1E was resistant to trametinib, selumetinib, RDEA119, docetaxel and 17-AAG, High expression of UBE2C was resistant to masitinib, and Low expression of ERO1B made the EC more sensitive to FK866. We constructed an EC risk score model composed of 8 DEGs and gene resistance analysis, which can provide reference for prognosis prediction, diagnosis and treatment of the EC patients.

## Introduction

Esophageal cancer (EC) is a major health problem worldwide, and its incidence rate is rising rapidly. Because of its poor prognosis, it has also become the sixth leading cause of cancer death in the world^1^. Esophageal cancer is mainly divided into esophageal squamous cell carcinoma (ESCC) and esophageal adenocarcinoma (EAC). The former accounts for nearly 90% and is the main subtype in Asia and East Africa. However, recent epidemiological studies show that the incidence rate of the EAC has increased 3~4 times, and its proportion is increasing. In most western countries,The EAC has exceeded the ESCC and has become the most important histological type of the EC^2,3^. Risk factors of theEC include smoking, alcohol, hot drink, nitrosamine intake, genetic factors, Barrett's esophagus, reflux esophagitis (RE) and obesity^4^. Although much progress has been made in the diagnosis and treatment of the EC, the mortality of patients with the EC is between 15 and 20%, ranking the fourth in all cancer mortality^5^.

Multiple gene mutations are involved in the EC cases. Using high-throughput sequencing technology to analyze the comprehensive mutation directory, it is found that there are extensive genomic changes in tumors. Gene changes are usually accompanied by abnormal expression, which plays an increasingly important role in the early diagnosis and prognosis evaluation of the EC^6^. At present, some gene expression products are used as markers for the diagnosis and prognosis of the EC. For instance, more than 83% of the ESCC contain somatic mutations of TP53. The abnormal expression of TP53 in the ESCC is also one of the risk factors of the EAC. TP53 point mutations are common in adenocarcinoma and squamous cell carcinoma^7^. In addition, many genes controlling the cell cycle are also overexpressed in the ESCC. For example, CDK4 / CDK6 account for 23.6%, MDM2 account for 5.7% and CCND1 account for 46.4%, indicating that the above factors are involved in the development of the ESCC^1^. The expression of BTG3 in tumor tissues of the EAC patients was significantly lower than that in adjacent normal tissues, and was linked to T, N, M and tumor stage^8^. Therefore, it is urgent to widely identify the genomic abnormalities of the ESCC and clarify its molecular basis, so as to improve the early diagnosis assess and reduce the mortality of the EC.

In recent years, the rapid progress of gene chip technology and bioinformatics has provided convenience for the exploration and mining of key cancer genes^9^. However, integrating multiple tumor markers to improve the accuracy of tumor prognosis has traditionally been a difficult problem in its clinical application. Therefore, this study constructs a prognostic risk model by integrating the mRNA and clinicopathological feature data of the EC samples in the Cancer Genome Atlas (TCGA) database, and looks for biomarkers for patients with the EC with poor prognosis, so as to provide a strong theoretical basis for predicting the prognosis and treatment decision-making of the EC.

## Materials and methods

### Data acquisition

The RNA sequencing data sets of 11 normal esophageal tissues and 159 esophageal cancer specimens and the corresponding clinical data were downloaded from the Cancer Genome Atlas Database (TCGA, http://cancergenome.nih.gov/). After integrating the original data through Perl script, the gene annotation file (Homo_sapiens.GRCh38.99)(https://asia.ensembl.org/index.html) was used to convert the Ensembl ID into gene symbol.

### Identification of differentially expressed genes (DEGs)

The gene expression matrix was further analyzed with the R language "edgeR" package to identify DEGs in esophageal cancer^10^. The expression amount of genes duplicate names is averaged, and the non expressed genes are excluded. After genome correction, the variance of the normal group and tumor group was calculated. If the inter-group variance is greater than the intra-group variance, the gene is retained for further difference analysis. FDR <0.05 and |log FC| >2 were used as the inclusion criteria of differentially expressed genes (DEGs).

### Enrichment analysis

Gene Ontology (GO) and the Kyoto Encyclopedia of Genes and Genomes (KEGG) were enriched and analyzed by "clusterProfiler, pathview" software package^11,12^. GO consists of Biological Process (BP), Cellular Component (CC) and Molecular Function (MF). The first 10 enrichment pathways of GO and KEGG were selected respectively. *P* < 0.05 was considered statistically relevant.

### Screening of prognosis related genes and construction of prediction model

Clinicopathological data and differential expression of DEGs in 159 patients with EC were combined. Kaplan-Meier and univariate Cox regression analysis were used to identify OS related genes from differentially expressed DEGs in patients with the EC. Venn plots were used to visualize the intersection of prognostic genes generated by the two methods and further incorporated into multivariate Cox regression analysis. Finally, eight DEGs with significant prognostic value were obtained and the risk scoring formula was established. The risk score formula is generated by the integral of 8 differentially expressed DEGs and weighted univariate Cox regression coefficients. According to the risk score formula, taking the median risk score as the dividing point, each EC patient was divided into low-risk and high-risk groups. Kaplan-Meier analysis and log rank test were used to compare the differences of OS between high-risk and low-risk groups. Then, univariate and multivariate Cox regression analysis was performed to confirm the prognostic significance of the risk scoring system after adjusting other clinical variables. Survival dependent receiver operating characteristic (ROC) curves were used to evaluate the sensitivity and specificity of prognostic models.

### Construction of regulatory network of risk genes and drug sensitivity analysis

The miRNA expression profile of EC was extracted from TCGA database, including 185 EC tumor tissues and 13 normal tissue cases. Differentially expressed miRNAs in EC were screened by R language "edgeR" software package^10^. FDR <0.05 and |logFC| >1 were used as screening criteria. The bidirectional prediction function of mirDIP online tool was used to screen the regulatory relationship between risk genes and miRNA. Relationships with more than 5 prediction programs were included, and the PPI network was constructed by using Cytoscape software^13^. Then, GSCA online database was used to predict the relationship between risk genes and drug sensitivity in the EC.

### Tissue samples

Patients with esophageal cancer admitted to Huai’an First People’s Hospital from 2016 to 2017 were collected. 150 cases of esophageal cancer and adjacent tissues were surgically removed. They did not suffer from other tumors or diseases seriously threatening their life and health. They did not receive any form of antitumor treatment such as radiotherapy, chemotherapy and targeted therapy before operation. They were jointly confirmed as esophageal cancer by two or more pathologists. Clinical diagnostic data and pathological data were collected for subsequent analysis. It includes the information of clinical characteristics and the data of common diagnostic markers (TP53, BRCA1 and KI67) of esophageal cancer. All research experiments involving patient data were approved by the ethics committee of Huai’an First People’s Hospital (approval number:KY-2022-014-01).

### Immunohistochemical staining

The expression of UBE2C protein in tissues and cell lines was detected by immunohistochemistry. The tissue samples were fixed in 4% neutral formaldehyde for more than 48 hours, embedded in paraffin and cut into 4 pieces μ M slices, baking at 70 °C for 2h, dewaxing xylene I and II for 15min respectively, hydration with 70%, 80%, 90%, 100% gradient alcohol and double distilled water, put them into sodium citrate solution for high temperature and high pressure antigen repair, wash them with PBS twice, and incubate them with 3% hydrogen peroxide methanol solution at room temperature in dark for 30min to remove endogenous peroxidase activity. Drip non immune sheep serum and block them at room temperature for 1H, Add 1: 100 diluted UBE2C primary antibody working solution, incubate at 4 °C overnight, add secondary antibody after PBS washing for 3 times, incubate at 37 °C for 30min, DAB reagent will develop color after PBS washing for 3 times, hematoxylin will stain the nucleus, dehydrate with conventional gradient alcohol, and seal with Canadian neutral gum after drying. Brownish yellow particles in the cells are positive cells, the proportion of positive cells ranges from 0 to 100%, and the staining intensity ranges from weak to strong. The staining results were negative (0-1), weak positive (1-2), moderate (2-3) and strong positive (≥ 3).

### Cell culture and immunohistochemical staining

Human esophageal cancer cell lines (kyse150, TE-1 and Eca109) and normal esophageal cell lines (HEEC) were donated by China Center for Disease Control and prevention. Logarithmic growth cells were digested with 0.25% trypsin and resuspended in complete medium. The cells were inoculated on glass slides and cultured in an incubator at 37 °C, 5% CO2 and sufficient humidity. After the cells reach sufficient density, take out the glass slide or cover glass and immerse it in PBS (pH 7.4) for 3 times. Then, the cells were immersed in 4% paraformaldehyde for 20 minutes, washed with PBS for 3 times, and then observed by immunohistochemistry.

### Correlation analysis between UBE2C and common clinical markers in esophageal cancer

GEPIA database was used to analyze the correlation between UBE2C and tumor markers (TP53, BRCA1 and KI67) in esophageal cancer. Clinicopathological data of 150 patients with esophageal cancer was collected and sorted out. The diagnostic results of tumor markers (TP53, BRCA1 and KI67) in 86 patients were complete and analyzed and counted by pathologists. Pearson test was used to analyze the correlation between genes. The ROC curve was used to analyze the diagnostic efficacy of gene expression in esophageal cancer.

### Statistical processing

Kaplan-Meier and Cox survival analyses were performed using "survival" and "survivminer" R packages(https://CRAN.R-project.org/package=survminer)(https://CRAN.R-project.org/package=survival). The "survivalroc" R package is used to draw the ROC curve to evaluate the ability of prognostic factors to predict the prognosis of patients(https://CRAN.R-project.org/package=survivalROC). All statistical analyses were performed using R language (version 3.6.3)(https://www.r-project.org/) and SPSS17.0 software. Bilateral test *P* < 0.05 showed that the difference was statistically significant.

### Ethics approval

All research experiments involving patient data were approved by the ethics committee of Huai’an First People’s Hospital (approval number:KY-2022-014-01).

### Statement

All methods were carried out in accordance with relevant guidelines and regulations and informed consent was obtained from all subjects.

## Results

### Identification of DEGs

**Figure 1:**
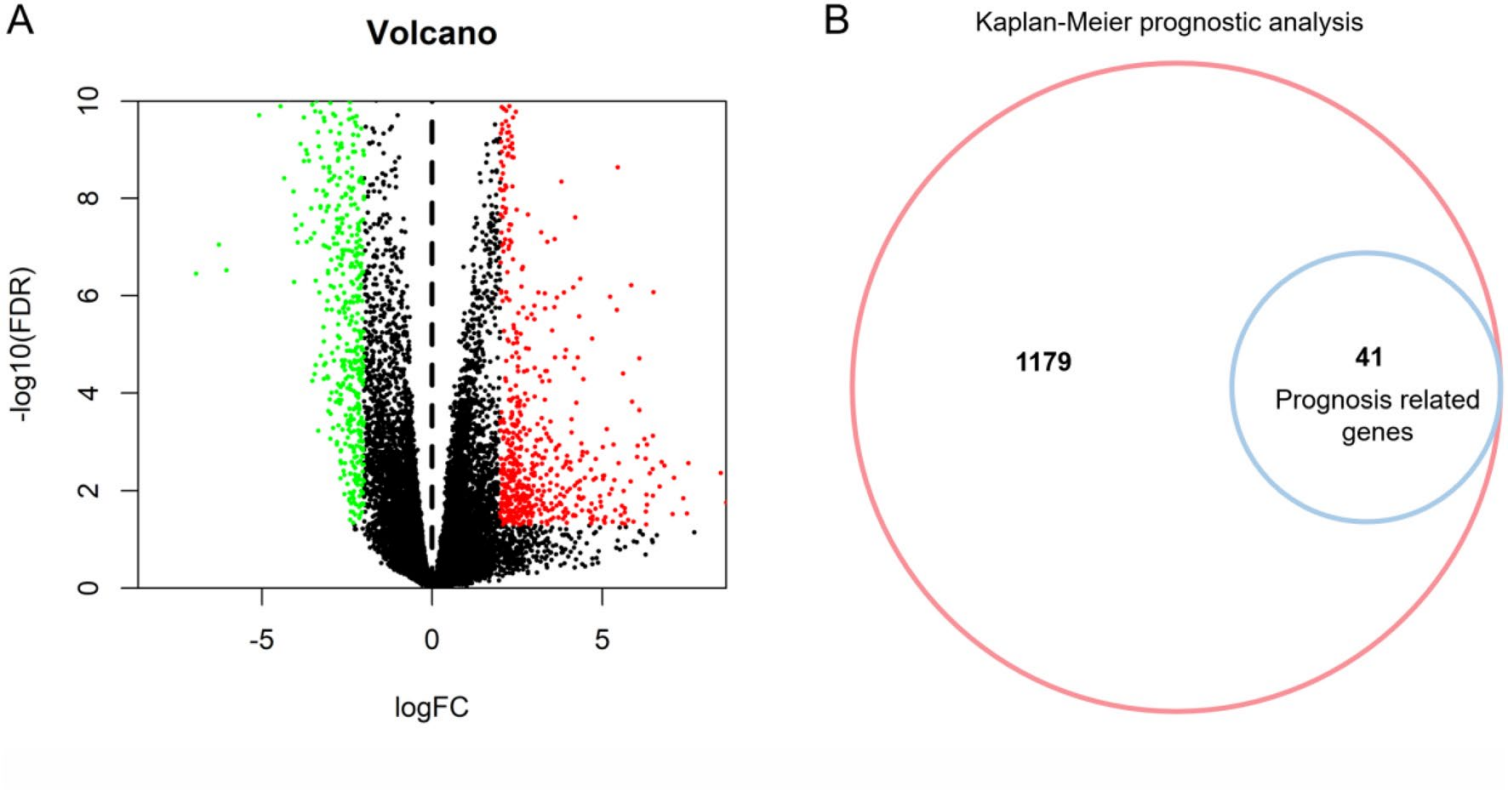
Differential gene analysis and prognosis analysis of esophageal cancer. (**A**) 1220 DEGs differentially expressed between the EC and adjacent tissues. Red dots represent differentially expressed up-regulated genes, green dots represent differentially expressed down-regulated genes, and black dots represent no significant difference in gene expression. (**B**) Kaplan–Meier (KM) prognostic analysis of DEGs. 41 genes of 1220 genes in the DEGs were associated with the prognosis of patients with esophageal cancer.

After data preprocessing, the RNA SEQ data of 159 EC specimens and 11 adjacent specimens were included. The volcano map (Fig. 1A) shows that 1220 DEGs are differentially expressed between EC and adjacent samples, in which the red dot represents significantly up-regulated genes, the green dot represents significantly down-regulated genes, and the black dot represents no difference genes.

### Screening of prognostic genes in EC

**Figure 2:**
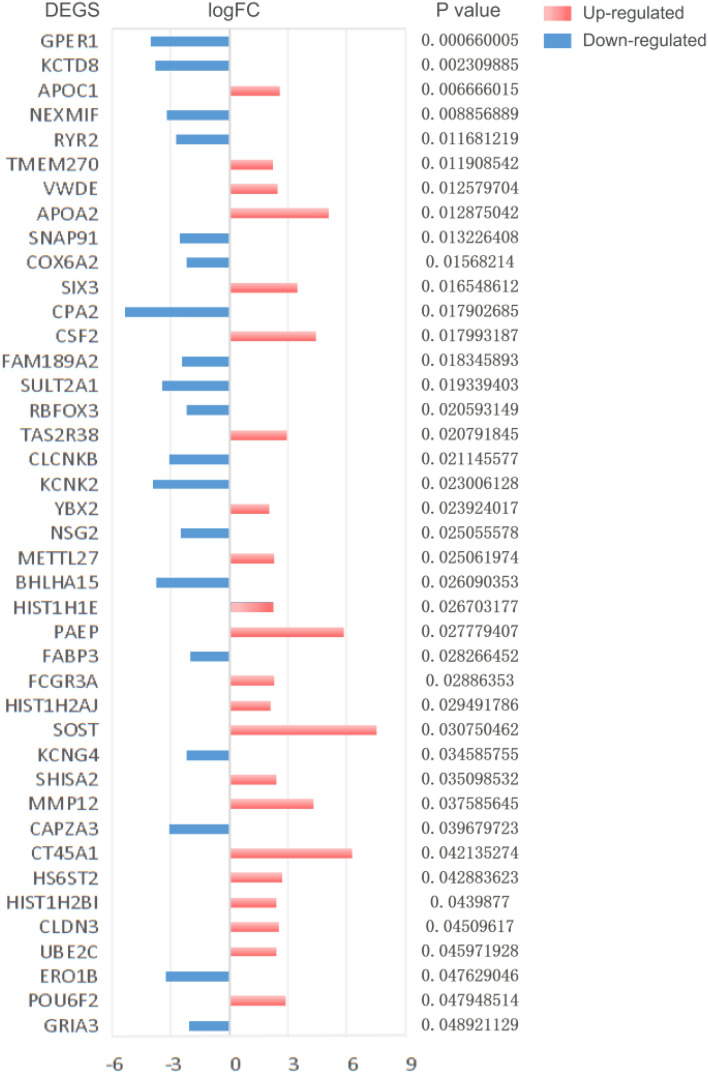
Up and down regulation of prognostically related DEGs in esophageal cancer. The red bar represents the up regulation of gene expression, the blue bar represents the down regulation of gene expression, and the length of the bar represents the value of |logFC|. The prognostic *p* value was shown on the right side of the gene.Up-regulated:APOC1,TMEM270,VWDE,APOA2,SIX3,CSF2,TAS2R38,YBX2,METTL27,PAEP,HIST1H2AJ,SOST,SHISA2,MMP12,CT45A1,HS6ST2,HIST1H2BI,CLDN3,UBE2C,POU6F2. Down-regulated:GPER1,KCTD8,NEXMIF,RYR2,SNAP91,COX6A2,CPA2,FAM189A2,SULT2A1,RBFOX3,CLCNKB,KCNK2,NSG2,BHLHA15,FABP3,KCNG4,CAPZA3,ERO1B,GRIA3.

Kaplan-Meier (KM) survival analysis was used to analyze the proteins related to the prognosis of the EC. The samples were divided into high expression or low expression (relative to median expression). The survival time and status of low expression group and high expression group were compared. Among 1220 DEGs, 41 proteins were associated with the prognosis of the EC (Fig. 1B). Among them, 22 genes were up-regulated and 19 genes were down regulated (Fig. 2).

### Functional enrichment analysis

**Figure 3:**
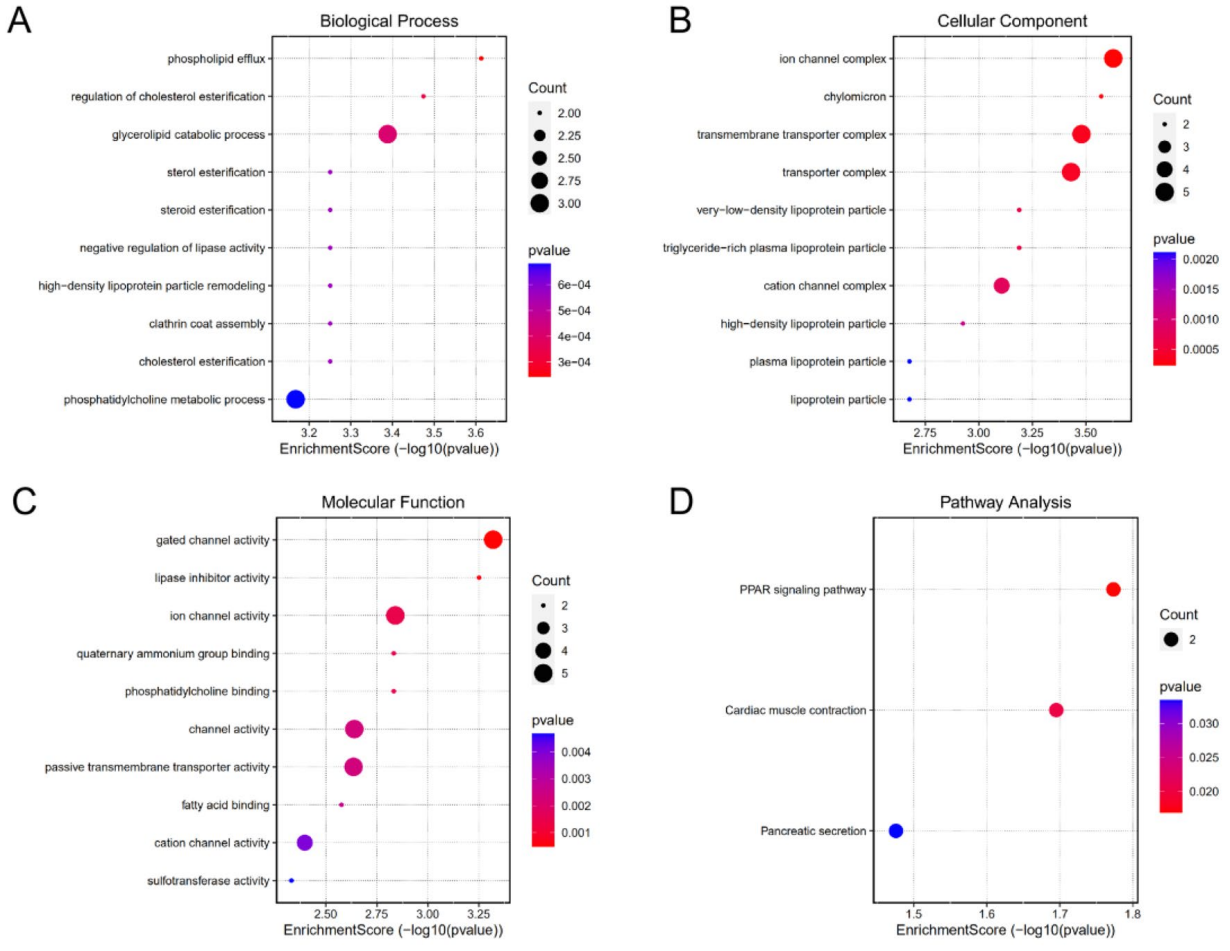
GO and KEGG enrichment analysis of 41 DEGs related to prognosis in EC. (**A**) Biological Process (BP). (**B**) Cellular Component(CC). (**C**) Molecular Function (MF). (**D**) Kyoto Encyclopedia of Genes and Genomes (KEGG). (According to the *p* value from small to large, the color of the circle increases from red to blue. The size of the circle indicates the count, that is, the number of genes.)

GO and KEGG pathway enrichment analysis was performed on 41 prognostic related DEGs. The results show that the GO analysis shows that the biological process (Fig. 3A) includes phospholipid flux, regulation of cholesterol estimation, glycolipid catabolic process, high-density lipoprotein particle remodelling, steroid estimation, sterol estimation, cholesterol estimation, crathrin coat assembly, negative regulation of lipase activity, Phosphoridylcholine metallic process. In cellular component (Fig. 3B), it includes ion channel complex, chylomicron, transmembrane transporter complex, transporter complex, very low density lipoprotein particle, triglyceride rich plasma lipoprotein particle, cation channel complex, high density lipoprotein particle, plasma lipoprotein particle and lipoprotein particle. The molecular function (Fig. 3C) includes gated channel activity, lipase inhibitor activity, ion channel activity, phosphotylcholine binding, quaternary ammonium group binding, channel activity, passive transmembrane transporter activity, fatty acid binding, location channel activity and sulfotransferase activity. Through GO analysis, it is found that DEGs play a role in lipid metabolism, channel complex composition and channel activity, and gene differential expression may lead to the disorder of the above biological functions of cells. KEGG pathway is mainly concentrated in PPAR signaling pathway, cardiac muscle contract and pancreatic secret (Fig. 3 D). Among them, previous studies believe that peroxisome promoter activated receptor (PPAR) signaling pathway has PPAR signal imbalance in a variety of cancers and focuses on a variety of metabolic pathways^14^.

### Construction and evaluation of prognostic model

**Figure 4:**
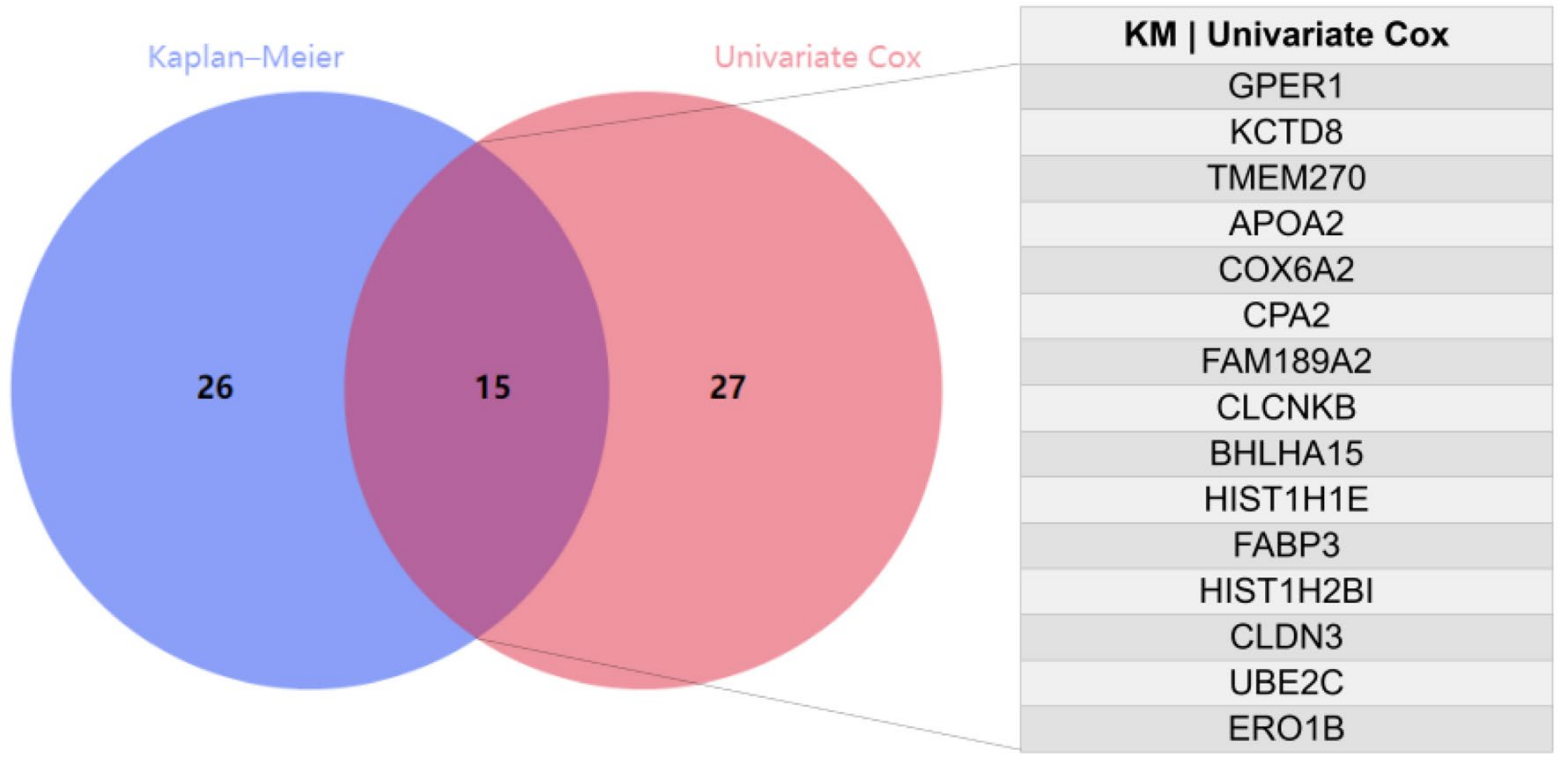
Venn diagrams for screening prognostic genes of the EC by Kaplan–Meier (KM) and univariate Cox analysis. A total of 15 common prognostic related genes (GPER1,KCTD8,TMEM270,APOA2,COX6A2,CPA2,FAM189A2,CLCNKB,BHLHA15,HIST1H1E,FABP3,HIST1H2BI,CLDN3,UBE2C and ERO1B) were found.

**Figure 5:**
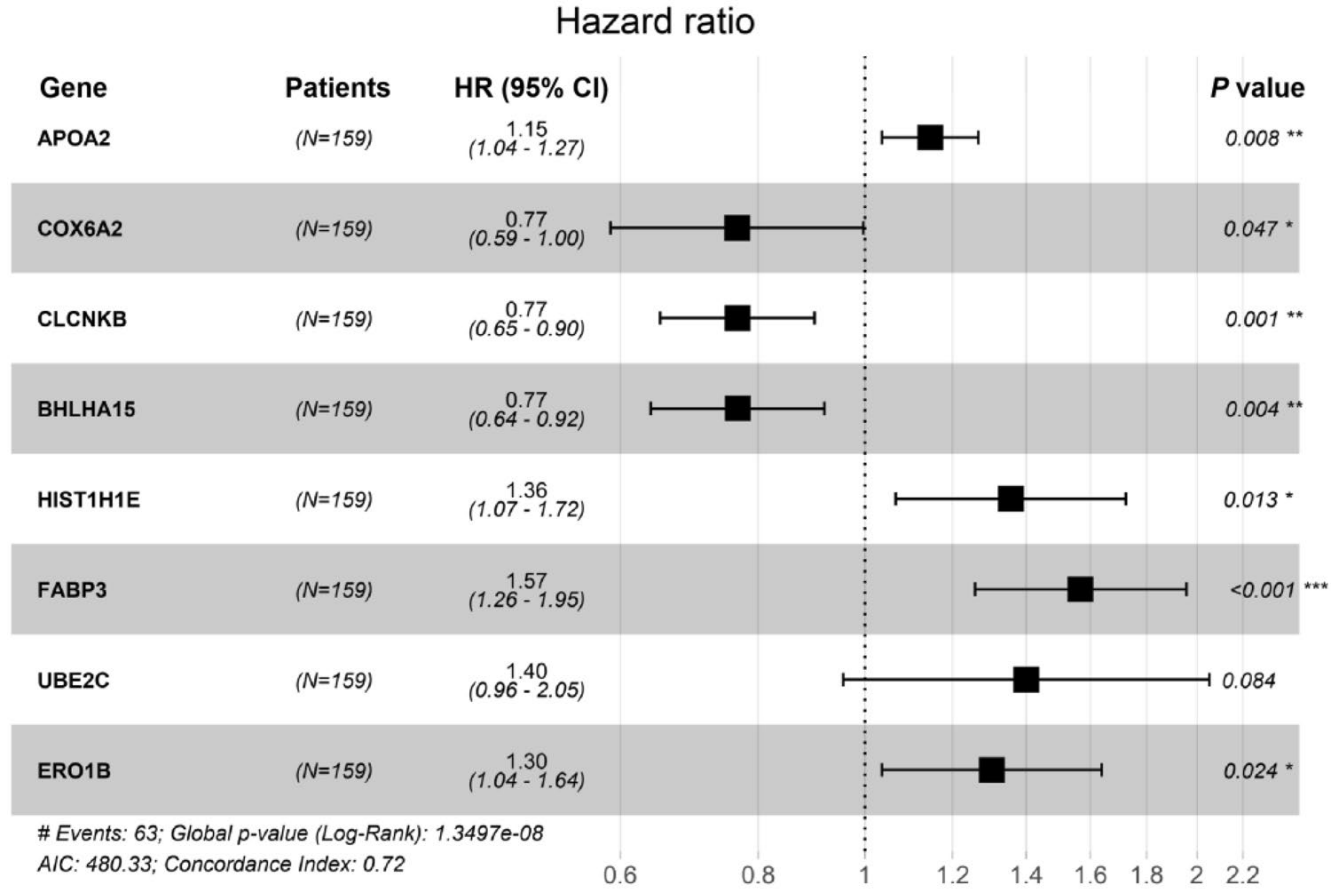
Forest maps of multivariate Cox regression analysis results. HR (Hazard Ratio) represents the risk coefficient of high expression group relative to low expression group. If HR > 1, the gene is a risk factor; If HR < 1, the gene is a protective factor; 95% Cl represents HR confidence interval. * *p* < 0.05, ** *p* < 0.01, *** *p* < 0.001.

**Figure 6:**
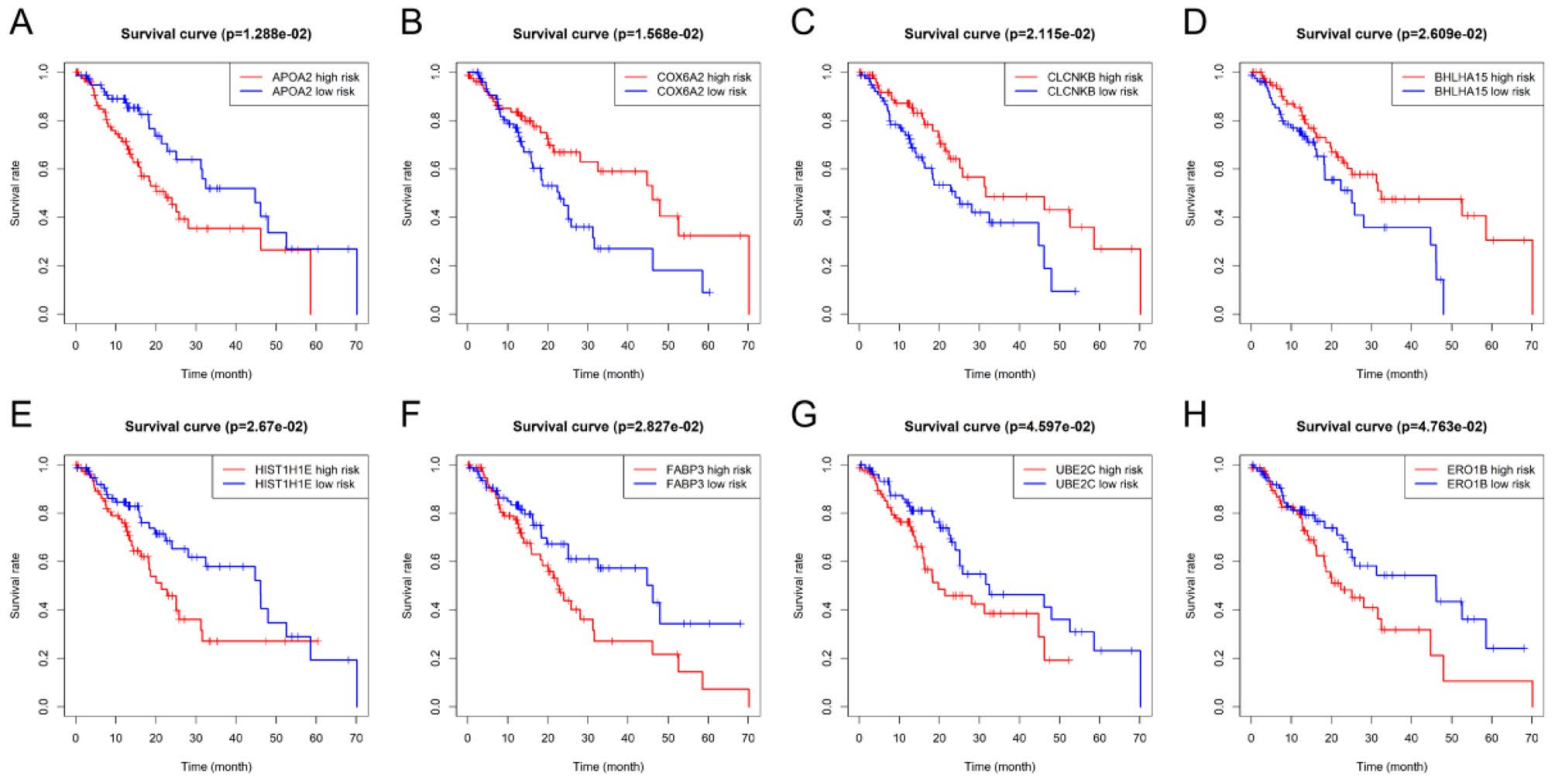
Kaplan–Meier (KM) survival curves of the EC patients with low or high individual risk score of eight genes. (**A**) Survival curve of APOA2. (**B**) Survival curve of COX6A2. (**C**) Survival curve of CLCNKB. (**D**) Survival curve of BHLHA15. (**E**) Survival curve of HIST1H1E. (**F**) Survival curve of FABP3. (**G**) Survival curve of UBE2C. (**H**) Survival curve of ERO1B. The red line represents the high-risk group and the blue line represents the low-risk group.

**Figure 7:**
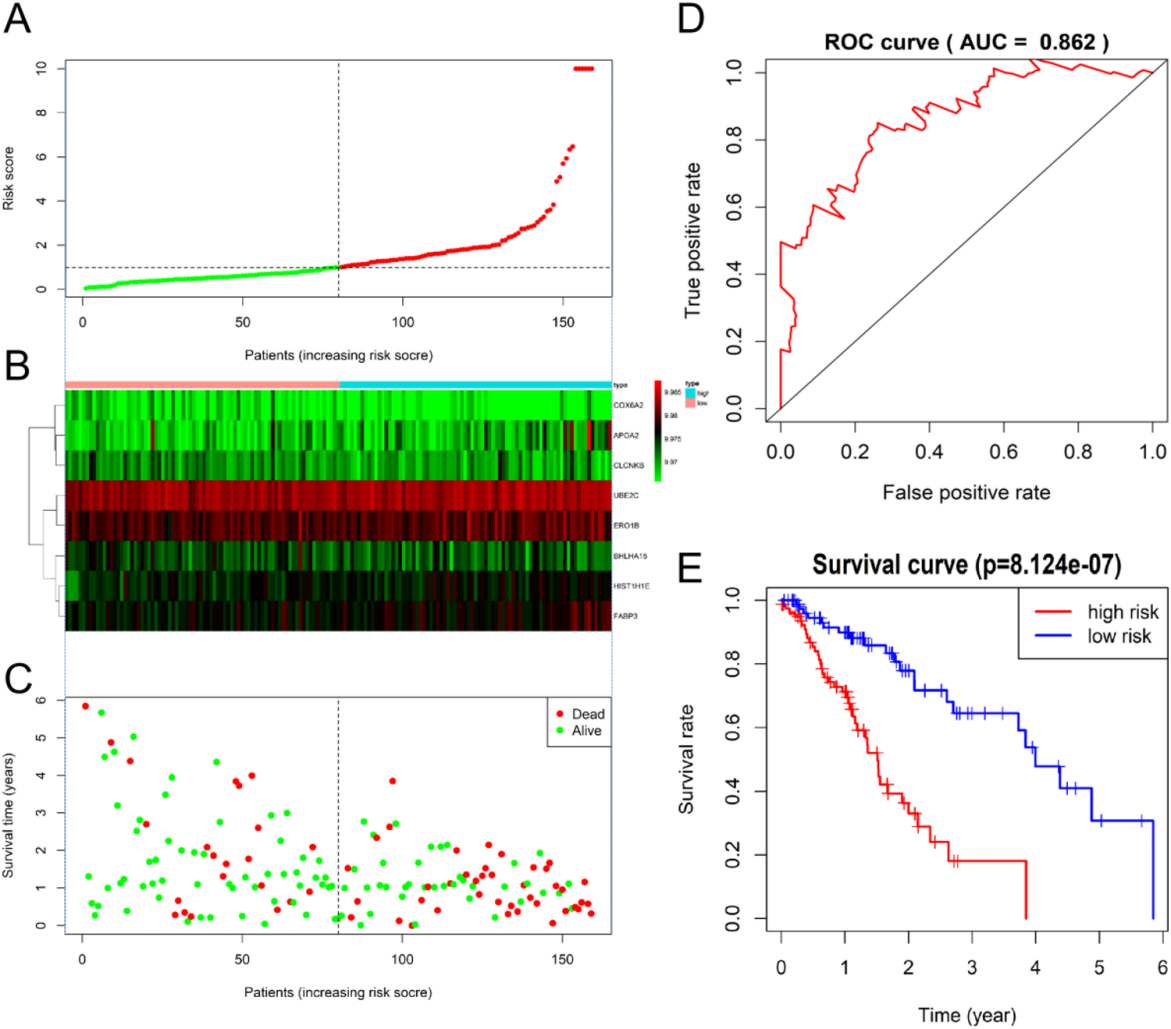
Performance evaluations of prognostic risk scoring model. (**A**) The patient's risk score, red indicates high risk and green indicates low risk. (**B**) Expression heat map of eight genes in high-risk group and low-risk group (Blue represents the high-risk group and red represents the low-risk group). (**C**) Distribution of survival status of patients in high-risk group and low-risk group (red indicates death and green indicates survival). (**D**) ROC curve of comprehensive risk scores of eight genes. AUC (area under curve) indicates the area below the ROC curve. The value is between 0 and 1. The higher the value, the better the prediction effect of the model. (**E**) KM survival curve of patients with the EC with low or high comprehensive risk score of eight genes.

The clinical follow-up data of 159 patients with EC were combined with 1220 DEGs. Univariate Cox analysis showed that 42 DEGs affected the prognosis of EC. Venn diagram shows 15 common prognostic related genes obtained by Kaplan-Meier (KM) and univariate Cox methods (Fig. 4). Then, multivariate Cox regression analysis was performed on these 15 candidate genes, and 8 DEGs (APOA2, COX6A2, CLCNKB, BHLHA15, HIST1H1E, FABP3, UBE2C and ERO1B) were significantly correlated with OS in patients with EC (Fig. 5). Among them, the HR values of APOA2, HIST1H1E, FABP3, UBE2C and ERO1B were greater than 1, which were potential risk factors. The HR values of COX6A2, CLCNKB and BHLHA15 were less than 1, which were potential protective factors. The difference of prognosis of eight genes was calculated by survival curve (Fig. 6). After integrating 8 genes and weighting their multivariable Cox regression coefficients, the risk score formula was obtained: (0.13592497×Expression of APOA2) + (-0.267351021×Expression of COX6A2) + (-0.26668478×Expression of CLCNKB) + (-0.265751714×Expression of BHLHA15) + (0.30430453×Expression of HIST1H1E) + (0.4497437×Expression of FABP3) + (0.336614943×Expression of UBE2C) + (0.264739114×Expression of ERO1B). According to the risk score formula, 159 patients with EC were given a risk value and divided into high-risk group and low-risk group with the median risk value as the cut-off value. The grouping results were visualized by prognostic feature distribution map (Fig. 7A), 8 DEGs expression profile heat map (Fig. 7B), patient survival map (Fig. 7C), ROC curve (Fig. 7D) and survival curve (Fig. 7E). KM survival curve showed that the survival rate of high-risk group was significantly lower than that of low-risk group (*P* = 8.124e-07).

### Evaluation of prognostic model as an independent prognostic factor

**Figure 8:**
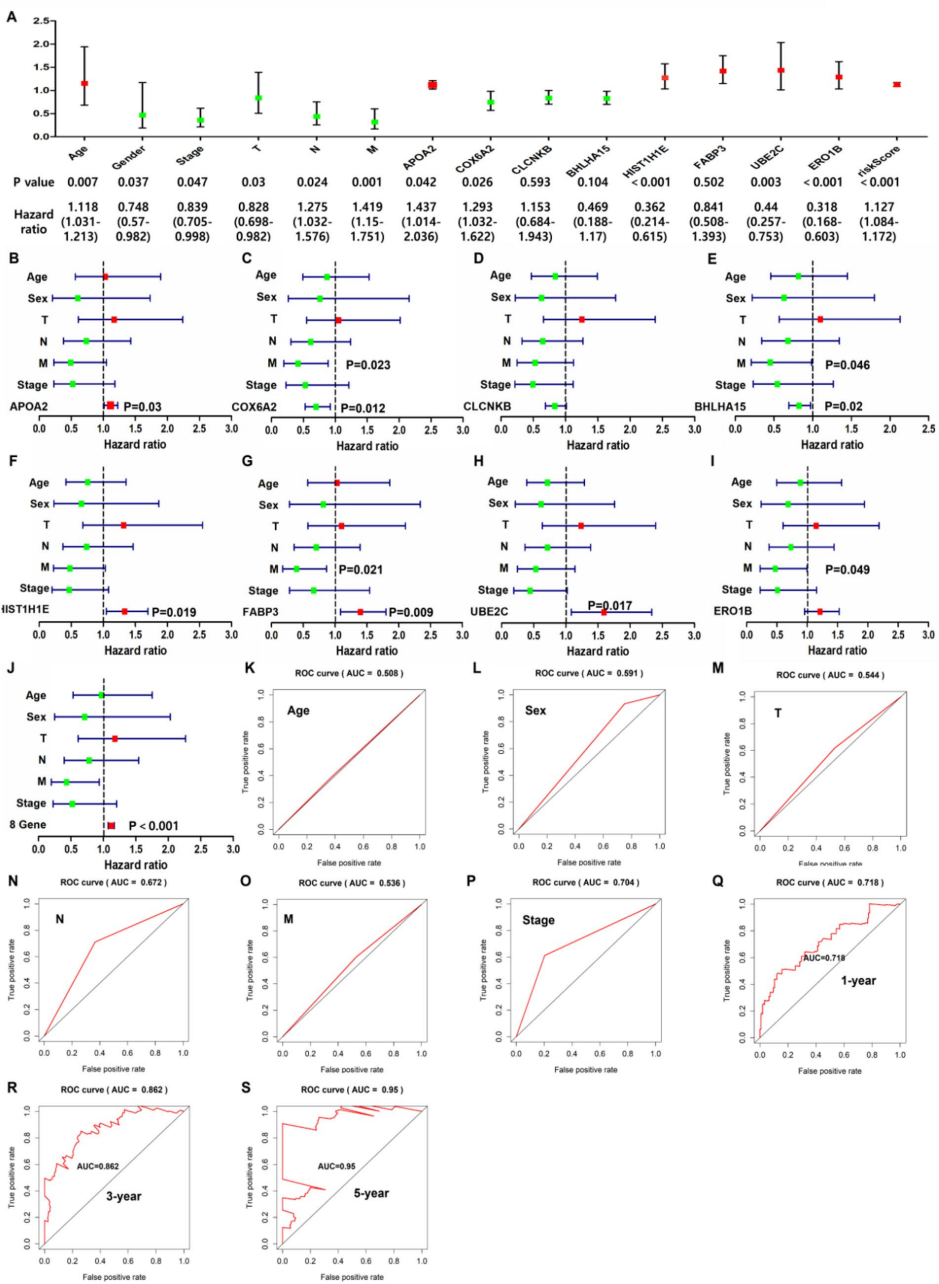
Evaluation of eight gene models as independent predictors. (**A**) ROC curve with comprehensive risk scores as a predictor. (**B**-**J**) Multivariate analysis of eight gene individuals and comprehensive risk scores involving patient characteristics. (**K**-**S**) ROC curve. The comprehensive risk scores of the eight genes under the 1-year, 3-year and 5-year curves were 0.718, 0.862 and 0.95.

The clinical characteristics of different individuals may affect their prognosis. Therefore, the calculated risk score and other clinical characteristics (age, gender, T, N, M and tumor stage) were included in univariate and multivariate Cox regression analysis. In univariate analysis (Fig. 8A), clinical characteristics (gender, age, T, N, M and tumor stage), APOA2, COX6A2, HIST1H1E, UBE2C, ERO1B and eight gene comprehensive risk scores were prognostic risk factors for patients with the EC (*P* < 0.05). In multivariate analysis (Fig. 8B-J), individual prognostic risk scores of APOA2 (*P* = 0.03), COX6A2 (*P* = 0.012), BHLHA15 (*P* = 0.022), HIST1H1E (*P* = 0.019), FABP3 (*P* = 0.009) and UBE2C (*P* = 0.017) were significantly correlated with prognosis. At the same time, the eight gene comprehensive risk score showed a stronger prognostic correlation (*P* < 0.001), indicating that the eight gene comprehensive risk score can be used as an independent predictor. ROC curve analysis is used to evaluate the prediction efficiency (Fig. 8K-S). The comprehensive risk scores of the eight genes under the 1-year, 3-year and 5-year curves were 0.718, 0.862 and 0.95 respectively, which was a better predictor than other characteristic factors.

### Regulatory network of risk gene-miRNA and drug sensitivity analysis

**Figure 9:**
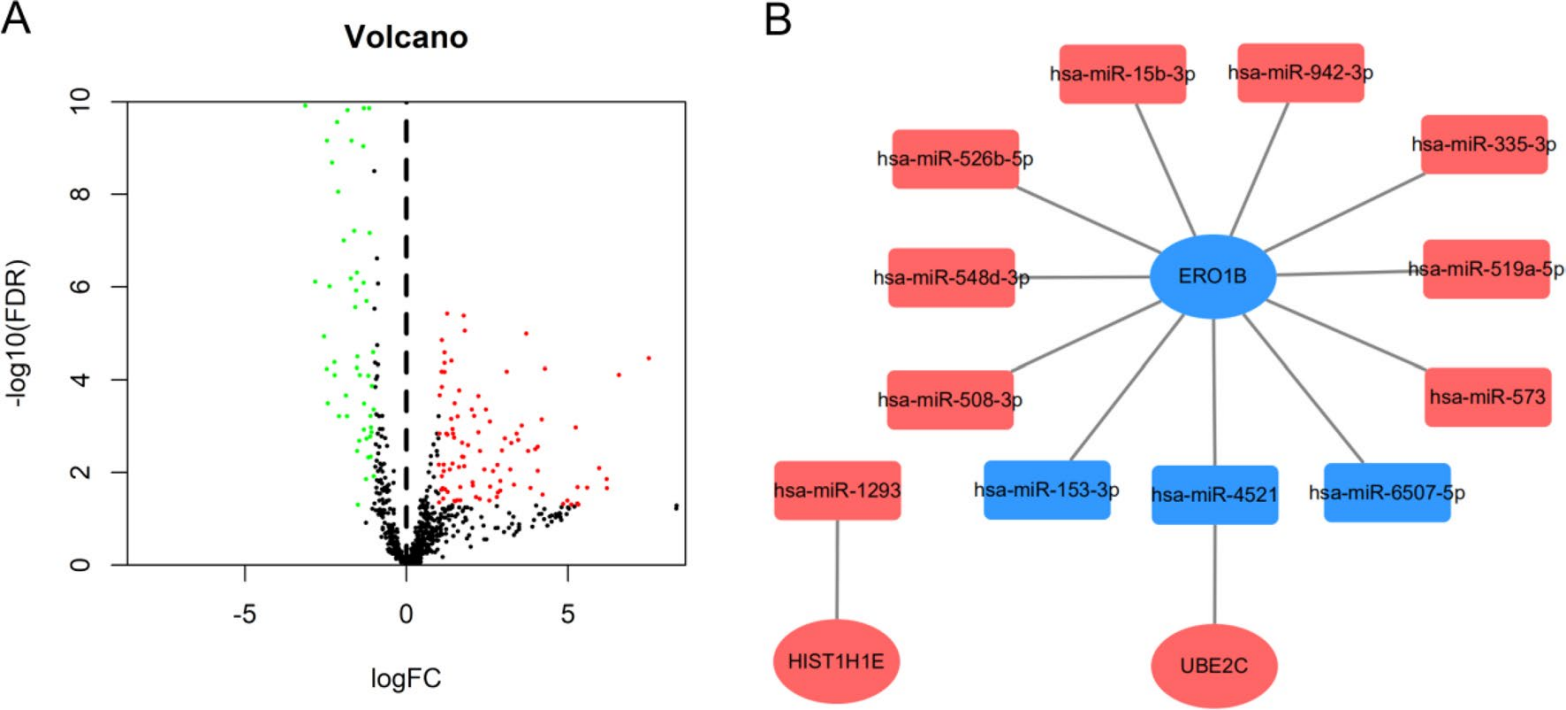
Construction of gene miRNA regulatory network. (**A**) Volcano map of differential expression miRNA screening. Green dots represent down regulated miRNAs and red dots represent up regulated miRNAs. (**B**) Regulatory network between risk genes and differentially expressed miRNAs. Red nodes represent up-regulated miRNAs and blue nodes represent down-regulated miRNAs.

**Figure 10:**
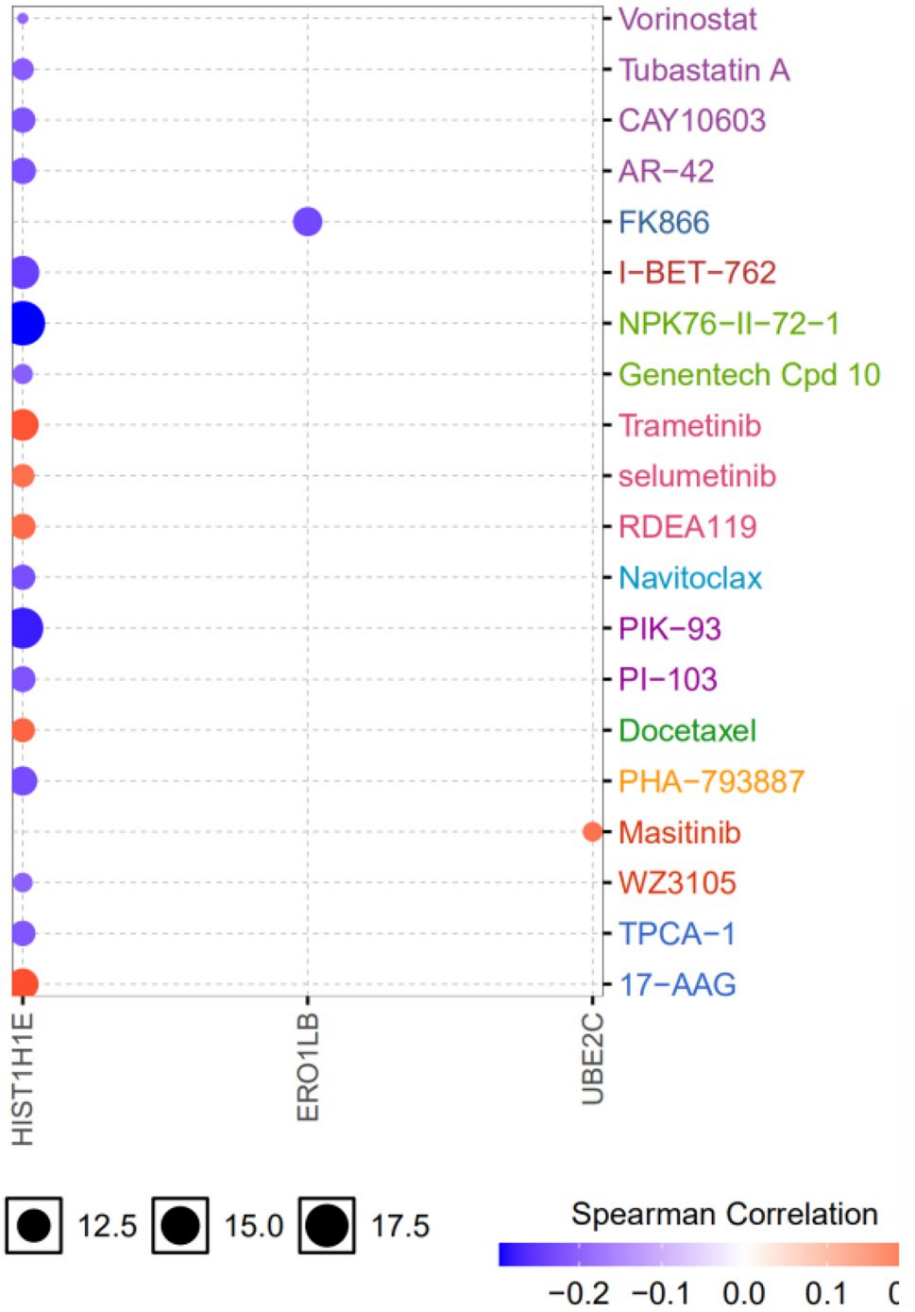
Potential drugs screened from GSCA database. Red nodes represent positive correlation and blue nodes represent negative correlation. The larger the node, the stronger the correlation. Positive correlation means that the high expression of the gene is resistant to drugs, and vice versa.

This study further collected and analyzed the miRNA expression profile data of the EC from the TCGA database. Among them, 98 up-regulated and 64 down regulated differentially expressed microRNAs were included (Fig. 9A). MirDIP analysis of the regulatory relationship between differential microRNAs and 8 prognostic mRNAs showed that 12 differentially expressed microRNAs had 13 potential regulatory relationships with 3 prognostic mRNAs (Fig. 9B). The results of drug sensitivity of risk genes showed that the high expression of HIST1H1E made tumor cells resistant to trametinib, selumetinib, RDEA119, Docetaxel and 17-AAG. The high expression of UBE2C makes tumor cells resistant to masitinib. The low expression of ERO1B makes the EC more sensitive to FK866 (Fig. 10).

### Expression of UBE2C in tissues and cells

**Figure 11:**
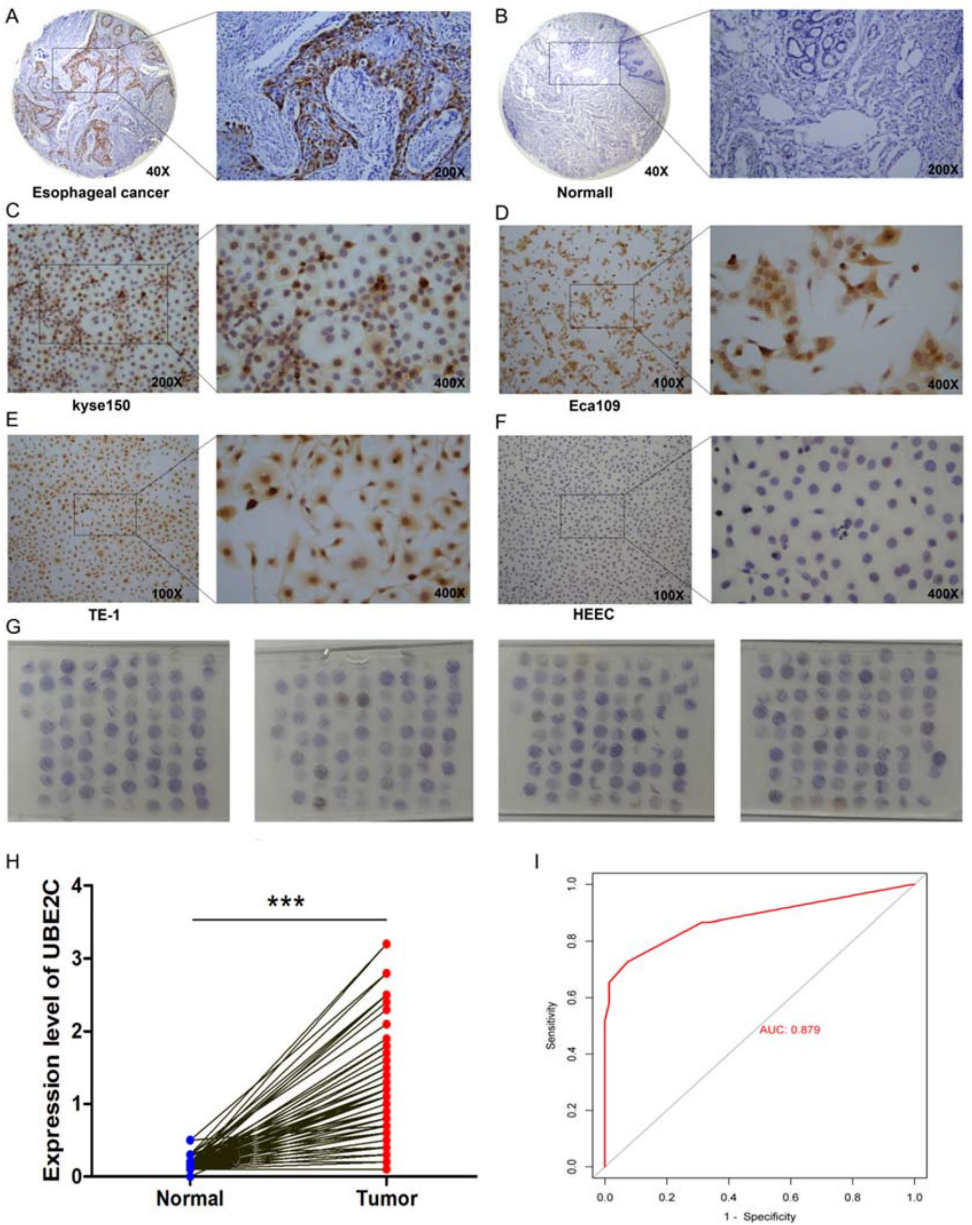
Immunohistochemical staining of UBE2C in the tissues and cell lines of the EC. (**A**) Expression of UBE2C in the EC. (**B**) Expression of UBE2C in normal esophageal tissues. (**C**) Expression of UBE2C in esophageal cancer cell line kyse150. (**D**) Expression of UBE2C in esophageal cancer cell line Eca109. (**E**) Expression of UBE2C in esophageal cancer cell line TE-1. (**F**) Expression of UBE2C in HEEC (normal esophageal cell line) . (**G**) Expression of UBE2C in the tissue microarray assay of 150 patients with esophageal cancer. (**H**) Differential expression analysis of UBE2C in cancer and adjacent tissues in the tissue microarray assay. Paired sample *t*-test was used to compare cancer and adjacent samples. ^***^P < 0.001. (**I**) Subject operating characteristic (ROC) curve analysis and area under curve (AUC) statistics were used to evaluate the ability of UBE2C to distinguish esophageal cancer from adjacent normal tissues.(AUC:0.879).

In immunohistochemical analysis, UBE2C was strongly expressed in esophageal cancer tissues (Fig. 11A) and negatively expressed in adjacent tissues (Fig. 11B). The expression was relatively strong in esophageal cancer cell lines (kyse150, TE-1 and Eca109) (Fig. 11C-E). The expression was negative in normal esophageal cell line (HEEC) (Fig. 11F). 150 pairs of cancer and adjacent tissues were concentrated and immunohistochemical expression chips were constructed (Fig. 11G). Subsequent statistical analysis found that the expression of UBE2C in esophageal cancer tissues was higher than that in adjacent tissues (Fig. 11H). ROC curve results showed that UBE2C had a good differential diagnosis ability for esophageal cancer (Fig. 11I). Correlation analysis between UBE2C expression and clinical factors showed that UBE2C expression was associated with tumor metastasis, and the positive rate of metastasis was high in patients with high expression (Table 1).

**Table 1:** Correlation between UBE2C expression and clinicopathological characteristics of 150 patients with the EC.

Factors	UBE2C expression		*P* value
	Low	High	
**Gender**			0.145729
Male	50	58	
Female	25	17	
**Age(years)**			0.870237
≥ 65	39	38	
< 65	36	37	
**Tumor size (cm)**			0.683091
≥ 5	14	16	
< 5	61	59	
**Lymph node metastasis**			2.15E-11***
Positive	17	58	
Negative	58	17	
**Differentiation**			0.404394
High	12	15	
Moderate	47	50	
Low	16	10	

***P < 0.001.

### Correlation between UBE2C and clinical indexes and diagnostic efficacy

**Figure 12:**
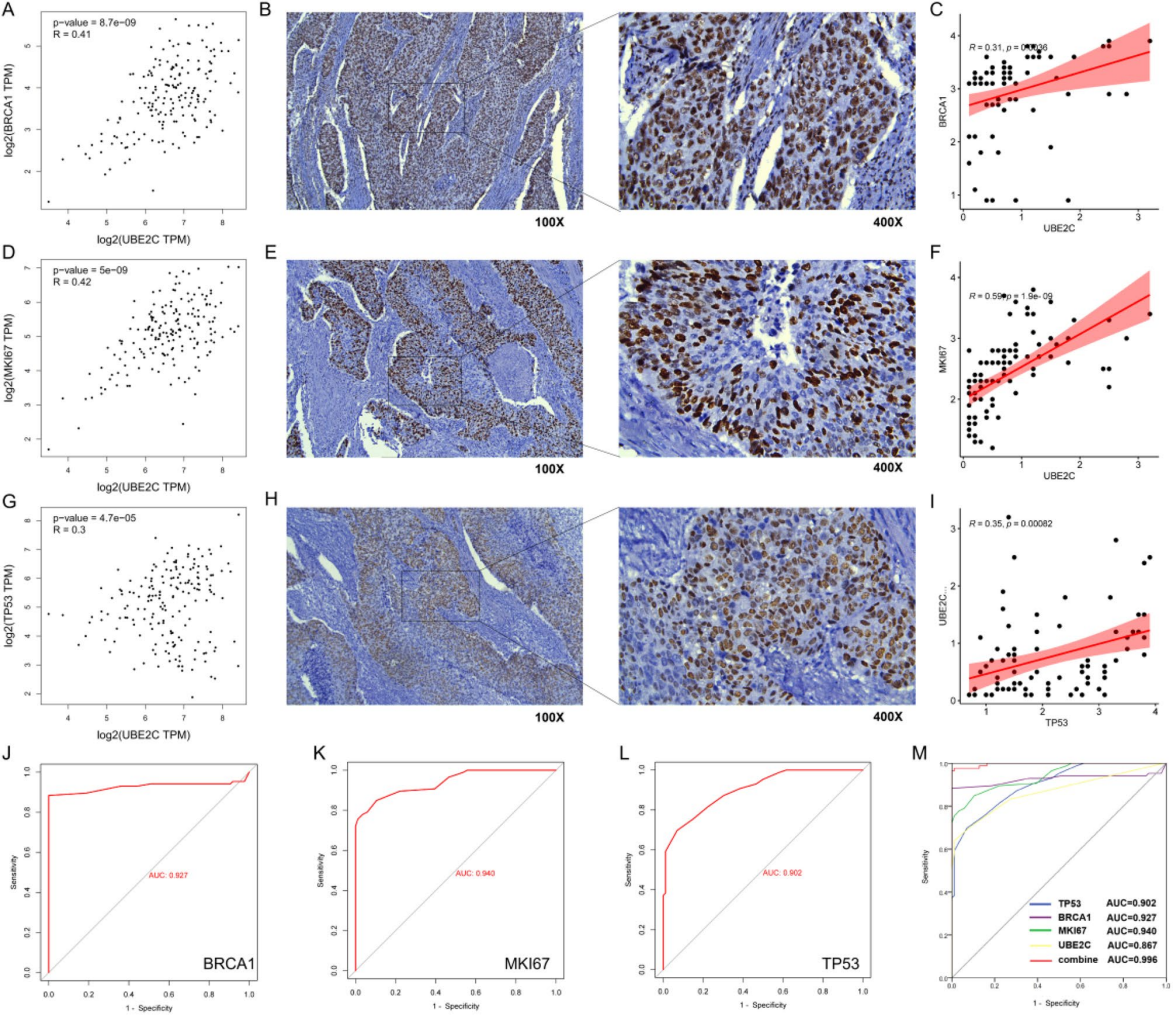
Correlation analysis between UBE2C and clinical markers and evaluation of combined diagnostic effect. (**A**, **D**, **G**) The correlation between UBE2C and clinical markers (BRCA1, KI67 and TP53) in esophageal cancer was analyzed by GEPIA database. R represents the correlation coefficient. (**B**, **E**, **H**) Expression of clinical markers (BRCA1, KI67 and TP53) in esophageal cancer. The brown part represents the target protein. (**C**, **F**, **I**) The correlation between UBE2C and clinical markers (BRCA1, KI67 and TP53) was analyzed according to the results of clinical immunohistochemistry. (J-L) ROC curve was used to analyze the diagnostic efficacy of clinical markers (BRCA1, KI67 and TP53) in esophageal cancer. (**M**) Efficacy of UBE2C combined with clinical markers (BRCA1, KI67 and TP53) in the diagnosis of esophageal cancer.

According to the results of database analysis, UBE2C was positively correlated with the expression of tumor markers (BRCA1, KI67 and TP53) in esophageal cancer (Fig. 12A,D,G). According to the analysis of clinical data, the expression of tumor markers (BRCA1, KI67 and TP53) is strong in cancer tissues (Fig. 12B,E,H). At the same time, UBE2C was positively correlated with the expression of clinical markers (BRCA1, KI67 and TP53) (Fig. 12C,F,I). ROC curve analysis shows that the areas under the curve of BRCA1, KI67 and TP53 are 0.927, 0.940 and 0.902 respectively (Fig. 12J,K,L). However, UBE2C combined with clinical markers (BRCA1, KI67 and TP53) calculated the highest area under the ROC curve, which was 0.996 (Fig. 12M).

## Discussion

EC is the main cause of cancer death, which is closely related to its innovative diagnosis and treatment. At present, the widely used prognostic factor is the clinicopathological characteristics. However, due to the genetic heterogeneity of the EC, it is difficult to accurately predict the prognosis through its clinicopathological features^15^. Smoking and gastroesophageal reflux diseases are the main risk factors for EC, which can cause different degrees of gene mutations in the EC^16^. Its heterogeneity leads to differences in key pathways and gene expression regulating tumor proliferation, migration and drug resistance. In addition,mechanisms of promoting cancer are complementary and staggered, which also poses a difficult problem for the diagnosis, treatment and prognosis of tumors^17^. At present, the exploration of molecular markers for the diagnosis, treatment and prognosis of the EC is not ideal. Even common tumor markers such as p53, Ki-67 and PD-1 need a comprehensive evaluation to provide reference for the diagnosis of EC, but these non proto cancerous molecular indicators are not ideal for the prediction of prognosis. With the popularization of high-throughput sequencing technology and the progress of bioinformatics analysis, gene data has entered the era of big data. TCGA database is the most comprehensive database to obtain molecular markers and clinical characteristics related to prognosis^18^. The establishment of a prognostic model based on the prognostic genes screened from the database and the exploration of its mechanism and drug resistance can serve as a theoretical basis for the clinical diagnosis and treatment of the EC.

In this study, DEGs were collected from TCGA database, and 41 prognostically valuable DEGs were screened by using esophageal cancer RNA-SEQ data and survival information. GO and KEGG enrichment analysis showed that these genes were mainly enriched in lipid metabolism, gated channel, transmembrane transport and PPAR signal pathway. Among them, PPAR is related to metabolic disorder and is an interesting drug target. However, the function of PPAR in cancer is controversial, which limits this research.Previous studies reported that PPAR- γ Agonists play an anti proliferative role in esophageal cancer^19^. On the other hand, PPAR was inhibited γ Activity leads to decreased invasiveness of esophageal cancer cells^20^. Therefore, this pathway is an entry point for the follow-up study of the EC. Eight key DEGs (APOA2, COX6A2, CLCNKB, BHLHA15, HIST1H1E, FABP3, UBE2C and ERO1B) were selected by univariate and multivariate Cox regression analysis to construct the prognostic risk score model of the EC. Among them, the HR values of APOA2, HIST1H1E, FABP3, UBE2C and ERO1B are greater than 1, which are potential risk factors. The HR values of COX6A2, CLCNKB and BHLHA15 are less than 1, which are potential protective factors.

Apolipoprotein A2 (APOA2) is the main component of high-density lipoprotein, which is composed of 77 amino acids and mainly circulates in the blood as a dimer^21^. The change of APOA2 ATQ/AT subtype concentration caused by abnormal treatment of APOA2 homologous two terminal C terminal can be used to distinguish between early stage pancreatic cancer and high risk pancreatic cancer patients and healthy control group^22^. APOA2 is maladjusted in ovarian cancer. It is an independent classification standard of malignant ovarian tumors and can be used as a biomarker of ovarian tumors^23^. Consistent with this study, APOA2 is up-regulated in the EC. It is a high risk gene and may become a marker to predict the prognosis of patients with the EC. Cytochrome c oxidase subunit 6A2 (COX6A2) is one of the nuclear coding polypeptide chains of cytochrome c oxidase. COX6A2 is expressed only in skeletal muscle and heart^24^. In this study, the expression of COX6A2 was down regulated in the EC. Patients with EC with low expression of COX6A2 had a poor prognosis, which may be a potential antitumor factor. Chloride channel protein CLC-KB (CLCNKB) belongs to the chloride channel family and plays an important role in regulating cell volume, signal transduction and cross epithelial transport. The specific gene expression of renal tumor subtype/nephron segment detected by RQ-PCR showed that the tumor expression of CLCNKB gene was relatively low^25^. We found that CLCNKB was also low expressed in the EC, and its low expression predicted a poor prognosis. A class of basic helix protein 15 (BHLHA15) plays a tumor inhibitory role in mice, and is down regulated in pancreatic cancer cell lines, and is related to differentiation^26^. BHLHA15 is a novel nuclear marker for acinic cell carcinoma of the salivary gland^27^. Similarly, the down-regulation of BHLHA15 expression in the EC can be used as a low-risk gene to predict the prognosis of patients. Histone Cluster 1, H1e (HIST1H1E) binds to the linker DNA between nucleosomes, which is necessary for nucleosome chain condensation to synthesize high-order structural fibers.HIST1H1E acts as a tumor suppressor. Because its overexpression inhibits the viability, colony formation, S-phase arrest, migration and invasion of lung cancer cells^28^. However, we found that HIST1H1E is highly expressed in EC, and the prognosis of patients with high expression is poor, and its gene function needs to be further verified. Fatty acid binding protein 3 (FABP3) is considered to play a role in the intracellular transport of long-chain fatty acids and their acyl CoA esters. Expression of fat acid binding protein-3 in gastrointestinal stromal tumors and its significance for diagnosis^29^. Similarly, FABP3 is low expressed in EC, and its high expression can be used as a marker of poor prognosis. Ubiquitin binding enzyme e2c (UBE2C) accepts ubiquitin in E1 complex and catalyzes its covalent connection with other proteins. UBE2C gene knockout in EC cells can significantly inhibit cell proliferation and induce apoptosis^30^. UBE2C mRNA expression can accurately distinguish EC and normal tissues. UBE2C directly interferes with the level of cycling B1 protein and affects the proliferation rate and cell cycle profile of ESCC cell line, indicating that UBE2C is involved in the key step of ESCC carcinogenesis^31^. Our study found that the expression of UBE2C in esophageal cancer tissues was higher than that in adjacent tissues, and it was also highly expressed in esophageal cancer cell lines (kyse150, TE-1 and Eca10). The differential expression of UBE2C in esophageal cancer and adjacent cancer makes it a reliable index for the diagnosis of the EC. Ero1 like protein β (ERO1B/ERO1LB), an oxidoreductase involved in the formation of endoplasmic reticulum disulfide bonds. Analysis shows that ERO1LB is involved in pancreatic cancer, and its expression in tumor tissues is lower than that in normal pancreatic tissues^32^. Similarly, ERO1LB is low expressed in the EC, and prognosis of patients with low expression is good, which may be used as a prognostic marker.

The survival analysis and risk score distribution of the above eight risk DEGs suggest that patients with low expression of COX6A2, CLCNKB and BHLHA15 genes and high expression of APOA2, HIST1H1E, FABP3, UBE2C and ERO1B genes have high risk scores and are more prone to poor prognosis (*P* < 0.05). By plotting the risk score distribution and Kaplan-Meier survival curve, it is proved that the prognosis of patients with a high risk score is worse than that of patients with a low risk score. The AUC of 1, 3 and 5 years proves that the model has good sensitivity and specificity in predicting the prognosis of the EC. At the same time, we also performed univariate and multivariate Cox regression analysis on the risk scores and other clinical predictors, which proved that the risk scores have an independent prognostic value and can be used as an independent prognostic predictor for patients with EC. TNM staging is an internationally recognized predictor of clinical prognosis. Although the risk scores (*P* < 0.001) are more advantageous than tumor staging from the perspective of univariate and multivariate Cox analysis, it can not explain that this model must be better than TNM staging. Because this model is still in the preliminary establishment stage and is a retrospective study with a small sample size, large-scale prospective clinical trial data are still needed to verify whether its prediction ability is better than TNM staging. After improving the basic experiment, it may be combined with TNM staging in clinical application to predict the prognosis of the EC patients in the future.

The change of drug resistance comes from the change of gene expression, and may affect the therapeutic effect of drugs^33^. Our study found that the high expression of hist1h1e was more sensitive to tubastatin A, vorinostat, CAY 10603, AR-42, I-BET-762, NPK76-II-72-1, Genentech Cpd 10, navitoclax, PIK-93, PI-103, PHA-793887, WZ3105 and TPCA-1, but resistant to trametinib, selumetinib, rdea119, docetaxel and 17-AAG. Tubastatin A^34^, vorinostat^35^, AR-42^36^ and navitoclax (ABT-263)^37^ all reported inhibitory effects on the proliferation of esophageal cancer cells. It is speculated that the high expression of HIST1H1E may play a role in drug sensitivity and drug action. In this paper, it is speculated that HIST1H1E is a high risk gene, and the prognosis of patients with high expression is poor. This study found that up-regulated miRNAs such as hsa-mir-15b-3p, hsa-mir-335-3p, hsa-mir-508-3p, hsa-mir-519a-5p, hsa-mir-526b-5p, hsa-mir-548d-3p, hsa-mir-573 and hsa-mir-942-3p regulated down regulated ERO1B. Combined with drug sensitivity analysis, the above differential miRNAs may enhance the drug resistance of FK866 by down regulating the expression of ERO1B. UBE2C is highly expressed in esophageal cancer and is associated with poor prognosis, which may be related to the mechanism of drug resistance to masitinib. The exploration of sensitive drugs based on abnormal gene expression can provide a theoretical basis for the development of clinical drugs.

Finally, in order to improve the clinical diagnosis rate, the correlation between the experimental results of UBE2C and clinical tumor markers (BRCA1, KI67 and TP53) was analyzed. It was found that UBE2C was positively correlated with the expression of tumor markers. At the same time, compared with the diagnostic efficiency of tumor markers alone, UBE2C combined with clinical markers has higher diagnostic efficiency, which reduces the misdiagnosis and missed diagnosis rate of patients to a certain extent.

## Conclusion

In conclusion, this study constructed a prognostic risk score model based on EC through univariate and multivariate Cox regression analysis, compared the value of this model with TNM staging in prognostic prediction, and found that the protein prognostic model was significantly better than the traditional model based on clinical characteristics. Based on the above advantages, protein prognosis model has been widely used in prognosis prediction of tumor patients^38^. The prediction performance of the model is stable and has an independent prognostic value. It can assist in providing reference for the individualized diagnosis and treatment of the EC patients.

## Data Availability

The datasets supporting the conclusion of this article are included within the article.

## Abbreviations

EC: Esophageal cancer
ESCC: Esophageal squamous cell carcinoma
EAC: Esophageal adenocarcinoma
RE: Reflux esophagitis
TCGA: The Cancer Genome Atlas
DEGs: Differentially expressed genes
GO: Gene Ontology
KEGG: Kyoto Encyclopedia of Genes and Genomes
ROC: Reciver operating characteristic
MF: Molecular function
CC: Cell components
BP: Biological process
OS: Overall survival
APOA2: Apolipoprotein A2
COX6A2: Cytochrome c oxidase subunit 6A2
CLCNKB: Chloride channel protein CLC-KB
BHLHA15: Basic helix protein 15
HIST1H1E: Histone Cluster 1, H1e
FABP3: Fatty acid binding protein 3
UBE2C: Ubiquitin binding enzyme e2c
ERO1B/ERO1LB: Ero1 like protein β
